# Consumer Psychology of Mysterious Consumption: Embracing Uncertainty through a Perception of Control

**DOI:** 10.3390/bs14050411

**Published:** 2024-05-14

**Authors:** Wei Ding, Seunghee Han

**Affiliations:** Department of Business Administration, Chung-Ang University, Seoul 06974, Republic of Korea; jeongmiyaa@naver.com

**Keywords:** consumer psychology, mysterious consumption, sense of control, uncertainty, random flight ticket

## Abstract

Mysterious consumption, characterized by product purchases without knowledge of their exact nature, is gaining popularity in the modern marketplace. In two online experiments, we examined how consumers’ psychological states, particularly their sense of personal control, influence their perception and intent to purchase mysterious products in the context of purchasing a random flight ticket. Study 1 demonstrated that when consumers experience low personal control, as opposed to high personal control, they are less likely to value the uncertainty inherent in random flight tickets, resulting in decreased purchase intentions. Study 2 revealed that introducing even limited choice options to random flight tickets can enhance consumers’ appreciation of uncertainty, thereby increasing their purchase intention. This effect was especially notable among those initially hesitant to embrace uncertainty, ultimately boosting their intent to purchase. These findings deepen our understanding of consumer psychology surrounding mystery consumption and provide practical insights for marketers seeking to tailor their products and marketing appeals to consumers’ psychological states.

## 1. Introduction

The popularity of mystery products has surged in recent years, spanning across various industries. Whether it is mystery boxes filled with random toys, beverages featuring undisclosed flavors, or even mystery travel packages, the trend has made its mark, captivating consumers worldwide. The craze surrounding mystery consumption is intriguing, considering that consumers generally find uncertainty discomforting. Indeed, consumers go to great lengths to avoid uncertainty, investing both time and money to attain certainty in their purchases. They devote significant time researching options, reading reviews, and comparing prices. They often opt for well-known brands with established reputations, even if they come with a higher price tag, and willingly pay extra for items’ guaranteed returns.

Yet, it is hard to deny that, at least for positive outcomes, uncertainty has an exciting and hedonically rewarding side. Incomplete information about an outcome can pique interest and curiosity. Anticipating how an event will unfold can be enjoyable. Surprise upon the reveal of an outcome can be fun. It appears that people understand these; people enjoy surprise, as evidenced by the prevalence of mystery consumption, whether in retail, the travel industry, or even when socializing. Mystery consumption represents a clear instance illustrating that consumers seek out uncertainty.

Thus, a more nuanced understanding of when consumers are more willing or less willing to embrace uncertainty is needed. We seek to investigate this in the context of mystery consumption. We propose that an individual’s psychological state, specifically feelings of personal control, can affect consumers’ perception of, and response to, uncertainty in mystery consumption.

Personal control, the sense that one can produce desired outcomes and prevent undesired ones [1], is a basic human need (e.g., [2]). As such, individuals try to achieve and maintain their personal control. A threatened or otherwise diminished sense of personal control triggers various perceptual, affective, and behavioral responses, aimed at restoring that sense of control (for a review, [3]). This suggests that consumers may respond to products and services quite differently when they feel a threat to their personal control. Indeed, research shows that consumer choices are meaningfully altered when they experience declines in feelings of personal control.

Drawing on this literature, we propose that consumers are more likely to embrace uncertainty in the context of mysterious consumption when they feel higher levels of personal control. Specifically, we predict that feelings of personal control will lead consumers to appreciate the uncertainty embedded in mystery products, resulting in higher purchase intention. This notion also enables us to predict that a product feature that provides consumers with a way to ascertain their sense of personal control will help consumers appreciate uncertainty more than they otherwise would.

We organize the remainder of this articles as follows. A brief review of prior research on personal control and positive perception of uncertainty leads to our predictions. Next, two experiments test our predictions and their proposed explanation, based on uncertainty appreciation. Finally, we discuss the theoretical and practical implications of the findings.

### 1.1. Theoretical Background

#### 1.1.1. Mysterious Consumption

Mystery consumption, a phenomenon whereby consumers purchase products without knowing their exact nature, has a long history, from Twister (vending machine-dispensed capsule toys) in the 1960s, to the craze of bubble-gum packs, baseball cards, and Pokémon cards in the 1990s. In the last decade, mystery product strategy was once again popularized and extended its reach to a wider range of settings, to luxury brands, travel agencies, fast food restaurants, and even museums. Some notable examples include toy manufacturers that sell collectable designer toys in blind boxes (e.g., Pop Mart), online booking sites that provide offerings of unknown nature (e.g., Hotwire for hotels), and subscription services for clothing and accessories (e.g., Stitch Fix).

Despite the burgeoning market prevalence of mystery products, the psychological mechanisms underpinning this consumer phenomenon remain largely uncharted. Prevailing literature predominantly concentrates on trends in the mystery product market, neglecting the fundamental psychological mechanisms that allure consumers toward, or deter them from, mystery products. Admittedly, mystery products are often coupled with attractive promotions such as discounted prices or limited-time offers, which at least partly explain their popularity. None of these, however, fully capture what is common to all of the examples outlined above, that the product is unknown and therefore uncertain at the time of purchase.

#### 1.1.2. Positive Sides of Uncertainty

The core characteristic of mystery products is the experience of uncertainty. Thus, mysterious consumption is driven in no small part by how consumers respond to uncertainty inherent in and unique to it.

Generally speaking, consumers favor certainty over uncertainty. Numerous research findings across economics, decision-making, psychology, and marketing literature showcase consumers’ aversion to uncertainty [4,5,6,7]. Indeed, certainty provides consumers with predictability of outcomes [5], lower transactional risk [8], reduced concern or anxiety [9], and lower cognitive load [10].

However, research findings highlighting the upsides of uncertainty do exist. First, uncertainty itself can be enjoyable. A recent study suggests that the experience of uncertainty can be exciting [11,12]. Consumers sometimes actively embrace uncertainty, as observed in behaviors such as delaying the discovery of outcomes (e.g., gender revelation of unborn babies), or taking measures to avoid spoilers that would reveal the ending [13,14,15,16]. Moon and Nelson (2020) [11] found that, compared to certain prospects, people consistently rate uncertain prospects higher in terms of anticipated enjoyment and interest. Buechel and Li (2022) [17] found that individuals prefer uncertain items over certain items when outcomes are horizontally differentiated (i.e., outcomes do not differ in objective superiority). For sure, uncertainty around positive outcomes provides consumers with the opportunity to speculate on a product’s potentially positive characteristics [18].

Relatedly, incomplete information can elicit curiosity and motivate people to expend effort to fill the gap [12] (Ruan et al., 2018). People invest more effort, time, and money to qualify for an uncertain reward than a certain reward when they focus on the process [19] (Shen et al., 2015). Similarly, uncertain promotions generate a greater consumer response than precise or certain discounts [20,21]. At least for positive outcomes, the literature acknowledges and provides evidence that uncertainty accompanies a pleasant anticipation of new information and heighted motivation. Additionally, resolving uncertainty can lead to positive hedonic experiences. For example, people believe that a surprise factor will enhance customer satisfaction with a product or a service [22] (Heilman et al. 2002).

Taken together, these streams of research suggest that uncertainty harbors an intriguing and hedonically rewarding aspect. The question is when, and under what circumstances, will these positive dimensions of uncertainty be recognized and appreciated. Needless to say, there are numerous dispositional and situational factors that can emphasize or de-emphasize the upsides of uncertainty. Current research focuses on the feelings of personal control individuals experience in the moment when they are presented with uncertainty.

#### 1.1.3. Threats to Personal Control and Predictable Consumption Experiences

Personal control is the extent to which an individual believes one can influence one’s outcomes and achieve goals (for a complete review, see [3]). Feelings of high personal control arise when an individual believes that their efforts can lead to desired outcomes, or prevent undesired outcomes. Conversely, feelings of low personal control arise when an individual believes that personal effort is irrelevant to the occurrence of any outcome.

Importantly, this conceptualization of personal control centers on the subjective perceptions of control, rather than on the objective levels of control individuals possess in reality. Additionally, perceptions of personal control can vary not only from person to person, but also across situations and over time [23]. That is, besides dispositional differences among individuals in their chronic sense of personal control, feelings of personal control fluctuate daily and throughout the lifespan. For instance, realizing that an important outcome is out of one’s hands, such as receiving a dreadful medical diagnosis or undergoing a major life transition, may cause threats to feelings of personal control. On the contrary, seeing things work out just as one predicted, such as receiving a good evaluation and following a daily routine, may promote feelings of personal control.

The desire to achieve and maintain personal control has long been regarded a fundamental motivating force in human life [23]. As such, it is one of the most crucial variables influencing psychological well-being and physical health [24,25,26]. Given its importance, individuals strive to maintain their sense of personal control, and a threat to personal control motivates them to consciously or unconsciously compensate for it, in order to shield themselves from a sense of helplessness and to keep agency in their lives [27].

One prominent means of compensation for individuals is through consumption activities (e.g., [28,29,30]). It has been demonstrated that consumers who feel threat to their personal control avoid uncertainty and seek predictable consumption experiences. For example, if they prefer familiar products instead of novel products because they were more likely to perceive the downsides of uncertainty involved in trying novel products [31,32]. They displayed an inclination towards products that require hard work, because those products helped them see the relationship between their effort and outcome; the observation of cause–effect contingency helped them experience order and structure in the world, which restores their sense of personal control [28].

#### 1.1.4. Uncertainty in Mystery Products

Mystery products, like novel products, entail uncertainties, perhaps to a greater degree. As a matter of fact, mystery products intentionally amplify uncertainties by concealing key information from consumers at the point of purchase. Moreover, mystery products, by their very nature, often lack a clear relationship between effort and outcome. Rather than direct effort leading to a predictable outcome, mystery products rely on chance, luck, or hidden factors. This can make them intriguing and unpredictable, but also challenging for consumers who seek a more straightforward experience of cause and effect.

We argue that these qualities of mystery products yield them not particularly attractive for consumers whose feelings of personal control are threatened, and who are thus are motivated to restore their sense of personal control. The uncertainties inherent in mystery products or their random structure, which defies predictable cause–effect relationships, may further undermine individuals’ feelings of control over their environment. Mystery products, therefore, are not likely to be the consumption choices that consumers seek when they sense a threat to their personal control. This leads us to predict that consumers’ feelings of personal control will shape their responses to mystery products. Specifically, we suggest that feelings of low personal control, compared to feelings of high personal control, will decrease consumer intention to purchase mystery products.

**Hypothesis** **1.** *Feelings of low personal control will decrease consumer intention to purchase mystery products, compared to feelings of high personal control.*

We further propose that the appreciation of uncertainty inherent in mystery products mediates the relationship between personal control and purchase intention. Uncertainty in products offers both opportunities and risks, and individuals’ psychological state can influence their perception. We posit that consumers’ perception of uncertainty in mystery products is shaped by their sense of personal control. Specifically, we predict that consumers with a low sense of personal control will be less inclined to appreciate the uncertainty embedded in mystery products, compared to those with a high sense of personal control. Consequently, this diminished appreciation of uncertainty is expected to reduce their intention to purchase mystery products. Therefore, we formally hypothesize the following.

**Hypothesis** **2.** *Appreciation of uncertainty will mediate the effect of personal control on consumer intention to purchase mystery products, such that feelings of low personal control will decrease appreciation of uncertainty compared to feelings of high personal control, subsequently resulting in reduced intention to purchase mystery products.*

#### 1.1.5. Choice as Vehicle for Perceiving Personal Control

One effective method for enhancing consumers’ sense of control in their consumption experiences is by providing them with opportunities to make choices. Research has shown that even minor choices can significantly bolster feelings of control, while the absence of choice diminishes it [33]. The connection between choice and perceptions of control is so ingrained in the human mind that even seemingly insignificant choices, such as selecting one’s own lottery ticket, can heighten the sense of personal control [34]. Given that the availability of choice can bolster consumers’ sense of personal control, we anticipate that mystery products offering choices could help consumers with low personal control perceive some level of control at the point of purchase and consequently, appreciate the uncertainty inherent in these products more than they otherwise would. This, in turn, could elevate their intention to purchase mystery products. Therefore, we formally hypothesize the following.

**Hypothesis** **3.** *The availability of choice will moderate the impact of personal control on uncertainty appreciation, such that the negative effect of low personal control on uncertainty appreciation will be reduced when choice is available for consumers compared to when choice is not available.*

**Figure 1:**
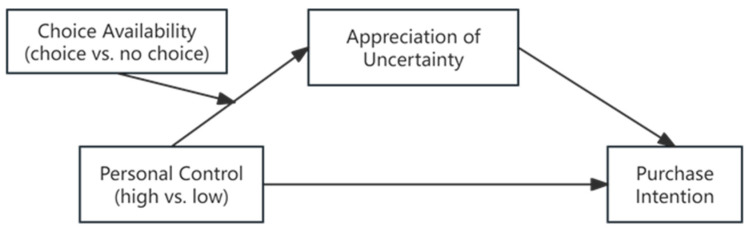
Conceptual model.

To recap, we aim to first demonstrate that experiencing low (versus high) personal control can prevent consumers from appreciating the uncertainties inherent in mystery products and, in turn, reduce their purchase intention. Furthermore, we explore the moderating effect of incorporating choice options in mystery products. We propose that introducing choice options to mystery products can enhance consumers’ sense of control, thereby increasing their appreciation of the uncertainties associated with such products. Our overall conceptual model is illustrated in Figure 1 below.

To date, despite the burgeoning market prevalence of mystery products, the psychological mechanisms underpinning this consumer phenomenon remain largely uncharted. The current research endeavors to take a small step forward. By highlighting the role of personal control in shaping consumers’ response to mystery products, we aim to advance our understanding of consumer psychology around mysterious consumption and provide useful insights for businesses marketing within this emerging market trend.

## 2. Study 1

A single factor experimental design with personal control (high or low) as the between-subject variable was employed to test Hypotheses 1 and 2. The mystery products we used to test our hypotheses are random flight tickets. As previously mentioned, the travel industry is one of the most prominent examples of mysterious consumption extending its reach to product categories that had not previously been subject to mystery product strategy. Over the past decade, the introduction of random flight tickets, which offer economically priced flight tickets to random destinations, has been witnessed globally (e.g., Australia’s Mystery Flights, America’s Hotwire, China’s Ctrip) and passionately welcomed by consumers [35]. Therefore, we decided to investigate this mystery product, which has captured the imagination of Generation Z and is expected to continue growing in popularity.

We predicted that consumers with feelings of low personal control would show reduced intention to purchase random flight tickets than those with feelings of high personal control (H1). Furthermore, we predicted that the appreciation of uncertainty would mediate the effect of personal control on consumer intention to purchase random flight tickets (H2). That is, feelings of low personal control will decrease the appreciation of uncertainty compared to feelings of high personal control, subsequently resulting in reduced intention to purchase random flight tickets.

## 3. Materials and Methods

Based on prior studies, we estimated a medium to small effect size of 0.40. A prior power analysis suggested a sample size of 200 to detect the effect when setting power at 0.80 and alpha at 0.05. With this as our target sample size, we recruited 230 participants from a Chinese online survey site. Out of 230 responses, we excluded 15 participants who failed to pass the embedded attention task and used the data from the remaining 215 participants. The mean age was 27.6 years and 62.3% of participants were female. Among the participants, 76.3% reported having no prior experience in purchasing random flight tickets.

Participants were randomly assigned to one of the two types of questionnaires, each designed to manipulate feelings of either high or low personal control. In the high personal control condition, participants first read an alleged news excerpt describing how people have more control over aspects of their health outcomes than previously thought. To strengthen the manipulation, participants were then asked to recall and briefly write about a life experience that illustrated the argument they read. In the low personal control condition, participants first read an alleged news excerpt describing how people have less control over aspects of their health outcomes than previously thought. To strengthen the manipulation, participants were then asked to recall and briefly write about a life experience that illustrated the argument they read. Next, all participants responded to the manipulation check questions that asked them to rate their perceived level of control over the situation they described in the writing task on two questions adapted from Min and Schwarz [32]: “I was in control of the situation” and “I had influence over what was happening”. Participants indicated how much they agree with each of the statements on a seven-point scale (1 = “strongly disagree”, and 7 = “strongly agree”). Participants’ response was averaged to form a personal control measure (Cronbach’s α was 0.818). Additionally, in order to check whether low versus high personal control manipulation affected participants’ mood in any systematic way, we have participants rate their mood using a scale adapted from Peterson and Sauber [36]. Participants indicated how much they agree with the statements like “Currently, I am in good mood” and “At this moment I am in bad mood” on a seven-point scale (1 = “strongly disagree”, and 7 = “strongly agree”).

**Figure 2:**
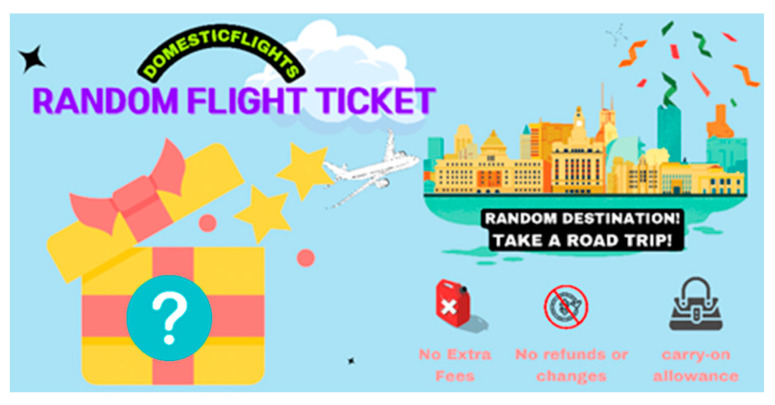
The banner summarizing the key points from the video in Study 1.

Next, in an ostensibly separate task, participants in both conditions viewed a 24-s video introducing a random flight ticket. This unskippable video explained that the product features a non-refundable flight ticket at $13, which includes one-way airfare for a single person to a random domestic destination, with a maximum carry-on baggage allowance of 7 kg. The video was followed by a print advertisement summarizing the key contents of the video (Figure 2). After watching the video and seeing the print advertisement, participants were asked to rate their purchase intention for the random flight ticket. Respondents reacted to items such as “The random flight ticket is a suitable option for me” and “I would like to purchase the random flight ticket” on a seven-point scale (1 = “strongly disagree”, and 7 = “strongly agree”). The Cronbach’s α was 0.935. They also reported how much they appreciated the uncertainty embedded in the random flight ticket, using a seven-point scale (1 = “strongly disagree” and 7 = “strongly agree”). Items were adapted from Ketelaar and Van’T and Thorbjornsen and Buijzen [18], and included items like “I like the fact that this random flight ticket leaves me something to guess about”, “I think it’s interesting that this random flight ticket doesn’t reveal everything”, and” Not knowing the destination in a random flight ticket is exciting to me”. The Cronbach’s α of this scale was 0.847. Finally, participants provided demographic information (e.g., gender, age, and purchase experience). The demographic variables did not predict significant variance in the dependent variable (nor did they interact with any other independent variable to predict the dependent variable).

## 4. Results

An independent sample *t*-test of personal control measure showed that participants in the high personal control condition (N = 114, *M* = 5.79, SD = 1.03) reported feeling more control than those in the low personal control condition (N = 101, *M* = 4.79, SD = 1.08; *t* = −6.97, *p*  < 0.001). However, there was no significant difference in the mood participants reported between the low (*M* = 4.12, SD = 1.45) and high personal control condition (*M* = 4.43, SD = 1.41, *t* = −1.57, *p* = *0*.117). Therefore, it was confirmed that the personal control manipulation made participants in the high control condition feel higher levels of personal control, compared to those in the low personal control condition, without making their mood any more positive.

An independent *t*-test was conducted to examine the difference in the purchase intention of a random flight ticket, based on personal control (high vs. low). The results revealed a significant effect of personal control on the intention to purchase random flight ticket (*t* = −2.52, *p* = 0.012). Specifically, participants in the low personal control condition (*M* = 4.96, SD = 1.23) reported lower purchase intention for a random flight ticket than those in the high personal control condition (*M* = 5.40, SD = 1.31). Thus, these findings provided support for Hypothesis 1.

Consistent with our Hypothesis 2, we found that participants in the low personal control condition reported lower appreciation of uncertainty than those in the high personal control condition (*M*_high_ = 5.09, SD = 1.04; *M*_low_ = 4.71, SD = 1.02; *t* = −2.69, *p* = 0.008). To test our prediction that the appreciation of uncertainty would mediate the effect of personal control on purchase intention, we conducted a mediation analysis using PROCESS Model 4 with 5000 bootstrap resamples [37]. Specifically, we examined the indirect effect of personal control (high vs. low) on purchase intention for random flight tickets via appreciation of uncertainty. The indirect effect was significant (B = 0.38, SE = 0.14, 95% CI: 0.10 to 0.65). That is, appreciation of uncertainty mediated the effect of personal control on purchase intention of random flight tickets. These results provided evidence for Hypothesis 2.

## 5. Discussion

Study 1 provided evidence for Hypotheses 1 and 2. The results demonstrated that consumers’ purchase intention for random flight tickets was lower for those who felt low personal control than for those who felt high personal control (H1). Our findings further revealed that differences in appreciation of uncertainty in high versus low personal control condition mediated the effect of personal control on purchase intention (H2). That is, when feeling a low level of personal control, compared to high personal control, consumers found it hard to appreciate the uncertainty inherent in the purchase of a random flight ticket, and were thus less willing to purchase them.

If our account of uncertainty appreciation as a critical underlying mechanism is accurate, one strategy to enhance the appeal of random flight tickets to consumers is facilitating their appreciation of the inherent uncertainty. We propose that introducing a product feature aimed at bolstering consumers’ sense of personal control could be an effective means of achieving this goal.

Choice has long been regarded as fundamental mechanism through which consumers exert control over their environment. Scholars have suggested that even minor choices can amplify or reaffirm the sense of control, while the absence of choice can erode it [33]. Decades of evidence from judgment and decision-making literature suggest that even when an option remains functionally identical, giving a choice imbues consumers with an “illusory” sense of control, a feeling that they are likely to achieve preferable outcome. For example, choosing one’s own lottery ticket, when all tickets have the same probability of winning, increased individuals’ perceptions of control [36,38]. These findings suggest that the availability of choice is a key product feature that can help boost consumers’ sense of control.

Expanding on these insights, in Study 2, we investigated whether incorporating a choice option as a new product feature would prompt consumers to perceive a greater sense of control at the point of purchase. We hypothesized that this enhanced perception of control would make consumers more inclined to appreciate the uncertainty of a random flight ticket, thereby increasing their intention to purchase.

## 6. Study 2

We employed a between-subjects experimental design involving a 2 (personal control: high vs. low) × 2 (choice availability: choice vs. no choice) framework to test Hypothesis 3. The no-choice condition used the exact same stimuli as Study 1, whereas the choice condition used a stimuli created by adding a choice option to the original stimuli.

We predicted this study to replicate the findings of Study 1 in the no-choice condition. However, in the choice condition, we expected to observe different pattern of results, particularly for consumers in the low personal control. Specifically, we precited that the availability of the choice option would help consumers in the low personal control condition to appreciate uncertainty in random flight tickets more so than they otherwise would, consequently increasing their intention to purchase them.

## 7. Materials and Methods

Based on a pretest, we estimated a medium to small effect size of 0.14. A prior power analysis suggested a sample size of 403 to detect the effect when setting power at 0.80 and alpha at 0.05. With this as our final target sample size, we recruited 431 participants from a Chinese online survey site. We excluded 24 participants who failed to pass the embedded attention task and used the data from the remaining 407 participants. The mean age was 28.95 years and 62.7% of participants were female. Among the participants, 76.9% reported having no prior experience in purchasing random flight tickets.

**Figure 3:**
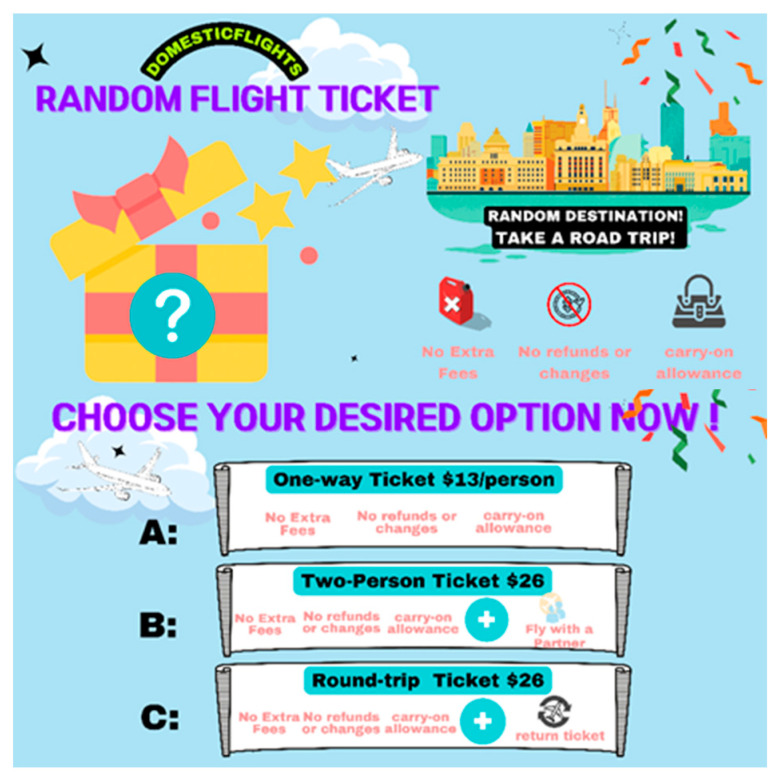
The banner summarizing the key points from the video in the Choice condition of Study 2.

Participants were randomly assigned to one of the four conditions created by crossing personal control factor (high vs. low) and choice availability factor (choice vs. no choice). All procedures remained identical to those in Study 1, with the exception that participants in the choice condition watched a modified advertisement for the random flight ticket. The advertisement included the same 24 s segment used in Study 1, followed by an additional 8 s segment. This extended segment explained that consumers had the option to choose from three ticketing options: a $13 one-way ticket, a $26 one-way ticket for two individuals, or a $26 round-trip ticket. Subsequently, participants were presented with a print advertisement summarizing the key points of the video (Figure 3). Participants in the no-choice condition viewed the exact same advertisement as those in Study 1. Upon completing the same series of questions measuring personal control, appreciation of uncertainty, and purchase intention as in Study 1, all participants were asked to answer manipulation check questions regarding choice availability.

## 8. Results

An independent samples t-test of the personal control measure showed that participants in the high personal control condition reported feeling more control than those in the low personal control condition (*M*_high_ = 5.50, SD = 0.90; *M*_low_ = 4.27, SD = 0.94; *t* = −13.397, *p* < 0.001). There was no significant difference in the mood participants reported between the low (*M* = 4.12, SD = 1.46) and high personal control condition (*M* = 4.27, SD = 1.29, *t* = −1.12, *p* = 0.263). Therefore, it was confirmed that personal control manipulation made participants in the high control condition feel higher levels of personal control compared to those in the low personal control condition, without making their mood any more positive. In addition, participants in the choice condition believed that they had choice options available to them, more so than those in the no-choice condition (*M*_choice_ = 5.34, *M*_no-choice_ = 4.56, *t* = −7.44, *p* < 0.001). Therefore, it was confirmed that the manipulation of choice availability was successful.

**Figure 4:**
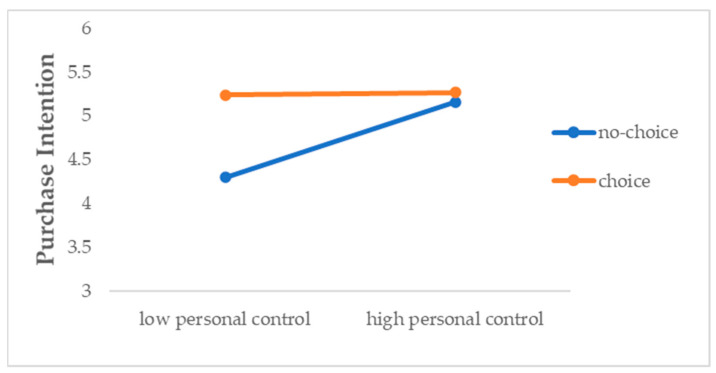
Choice availability moderates the effect of personal control on purchase intention.

We conducted a two-way ANOVA with personal control, choice availability, and their interaction as independent variables and purchase intention of random flight ticket as the dependent variable. The results revealed a significant main effect of both personal control (F (1, 403) = 18.78, *p* < 0.00, ⴄ^2^p = 0.045) and choice availability (F (1, 403) = 26.03, *p* < 0.00, ⴄ^2^p = 0.061). More importantly, these effects were qualified by a significant interaction between personal control and choice availability (F (1, 403) = 15.69, *p* < 0.00, ⴄ^2^p = 0.037). As shown in Figure 4, in the no-choice condition, which was the exact same setting as Study 1, the findings from Study 1 were replicated. That is, participants in the high (vs. low) personal control condition reported a greater purchase intention of the random flight ticket (*M*_high_ = 5.16, *M*_low_ = 4.30, *p* < 0.00). However, in the choice condition, there were non-significant differences between participants in the low versus high personal control conditions (*M*_high_ = 5.27, *M*_low_ = 5.24, *p* = 0.794). Additionally, comparison between no-choice and choice condition revealed that, when choice was made available, purchase intentions significantly and statistically increased for participants in the low personal control condition (*M*_no-choice_ = 4.30, *M*_choice_ = 5.24, *p* < 0.00).

We observed a similar pattern in participants’ appreciation of uncertainty. A two-way ANOVA revealed a significant main effect of personal control (F (1, 403) = 22.08, *p* < 0.00, ⴄ^2^p = 0.052), as well as a significant main effect of choice availability (F (1, 403) = 25.80, *p* < 0.00, ⴄ^2^p = 0.060). Again, these effects were qualified by a significant interaction between personal control and choice availability (F (1, 403) = 20.50, *p* < 0.00, ⴄ^2^p = 0.048). In the no-choice condition, which was in the same setting as Study 1, the findings from Study 1 were replicated (*M*_high_ = 5.18, *M*_low_ = 4.44, *p* < 0.00). That is, participants in the high personal control condition reported a greater appreciation of uncertainty compared to those in the low personal control condition. However, in the choice condition, there were virtually no differences between participants in the low and high personal control conditions (*M*_high_ = 5.23, *M*_low_ = 5.21, *p* = 0.904). Comparison between choice versus no-choice condition revealed that, when choice was made available, participants’ appreciation of uncertainty significantly and statistically increased for participants in the low personal control condition (*M*_no-choice_ = 4.44, *M*_choice_ = 5.21, *p* < 0.00).

To test whether the effect of personal control on purchase intention was moderated by choice availability and mediated by appreciation of uncertainty, we conducted a moderated mediation analysis using PROCESS Model 7 with 5000 bootstrap resamples [37]. The results indicated significant moderated mediation (B = −0.77, SE = 0.17, 95% confidence interval [CI]: [−0.12, −0.43]). Mediation by appreciation of uncertainty was significant in the no-choice condition (B = 0.75, SE = 0.11, 95% CI: 0.53 to 1.05), but was non-significant in the choice condition (B = 0.02, SE = 0.11, 95% CI: −0.19 to 0.25). Note that the sign of the indirect effect became non-significant in choice condition, suggesting that the availability of choice helped low personal control participants appreciate uncertainty as much as high personal control participants, resulting in a greater purchase intention than they would otherwise have had (Table 1). These findings provided evidence for Hypothesis 3.

## 9. Discussion

Study 2 replicated and extended the findings from Study 1. Once again, we observed that participants feeling low personal control, in contrast to those feeling high personal control, demonstrated less appreciation for the uncertainty associated with the random flight tickets, resulting in reduced intention to purchase them. However, when the random flight ticket was introduced with choice options for consumers, the difference in purchase intention between participants with low versus high personal control disappeared. As hypothesized, this effect was driven by increased appreciation of uncertainty for participants in the low personal control condition. In other words, the availability of choice prompted participants in the low personal control condition to appreciate the uncertainty of a random flight ticket, thereby increasing their purchase intention.

## 10. Overall Discussion

Mysterious consumption, product purchase without knowing its exact nature, is gaining traction in the modern marketplace. Current research examined consumer psychology of mystery consumption in the context of random flight ticket purchase intention. We specifically focused on one psychological factor, consumers’ sense of personal control, and examined how it affects their perception of the uncertainty inherent in purchasing a random flight ticket. The findings reveal that consumer responses to uncertainty are more complex and nuanced than commonly thought. Consumers are more willing to appreciate uncertainty involved in mysterious consumption when their sense of personal control is high rather than low. Study 1 demonstrated that consumers experiencing low levels of personal control are less likely to appreciate the inherent uncertainty of random flight tickets, resulting in decreased intention to purchase them, compared to those experiencing high levels of personal control. Study 2 demonstrated that adding choice options to random flight tickets, a feature that helps consumers feel more in control, can enhance their appreciation of the uncertainty inherent in such products. This effect is pronounced among individuals who initially showed less appreciation for uncertainty, consequently increasing their purchase intention.

### 10.1. Theoretical Contributions

Past literature on consumer judgment and decision-making has frequently emphasized widespread uncertainty and risk aversion [5,6]. However, recent studies have begun to shed light on the potential benefits of uncertainty [10,11,17,18]. We contribute to this literature by showing that consumers can indeed appreciate uncertainty, particularly within the context of random flight ticket purchases.

More importantly, we find that consumers’ appreciation of uncertainty can vary depending on their psychological state. Through a controlled experimental design, our research offers empirical evidence that sheds light on how consumers’ perception of personal control in the moment affects their tolerance for uncertainty and, consequently, their inclination to purchase random flight tickets. By emphasizing the impact of transient feelings of personal control on consumer choices, our research provides valuable insights that go beyond the traditional emphasis on personal control as a relatively stable individual trait.

Moreover, our research illustrates that consumers’ appreciation of uncertainty can be effectively managed through minor adjustments in product attributes. Specifically, providing choice options, even if limited, amplifies the sense of control for individuals feeling low personal control. This increase in personal control enables them to perceive uncertainty in a nearly as favorable manner as consumers with high personal control. These findings underscore the intricate interplay between consumers’ psychological state (such as personal control), their attitude towards uncertainty, and the specific features of products, all influencing consumer decision-making. They contribute to a more nuanced comprehension of how individual-level and situational factors interact to shape consumer responses towards uncertain products, such as random flight tickets.

### 10.2. Managerial Implications

Toy manufacturers that sell their product in blind boxes (e.g., Popmart), snack companies that introduce mystery flavors (e.g., Pringles), and booking sites that provide offerings of unknown nature (e.g., Hotwire for hotels) are just a few examples of a growing industry that introduces uncertainty about product selections at the time of purchase. Such products exist in the marketplace because consumers perceive value in them. While discounted prices, limited-time offers, or outsourcing choices to a third party may explain their popularity to some extent, none fully capture what is common to all of the examples outlined above: that the product is unknown and therefore uncertain at the time of purchase. Undoubtedly, mysterious consumption is largely driven by the inherent and unique uncertainty it presents.

In promoting mystery products, practitioners often emphasize the excitement and thrill of uncertainty. The findings from current research, however, suggest that this strategy may not be the most effective, as some consumers may not be inclined to appreciate such excitement and thrill. To promote mystery products more effectively and appeal to wider segments of consumers, marketers need to tailor their strategies based on their target consumers’ psychological states, particularly their sense of personal control in the moment.

Tailoring marketing appeals to consumers’ psychological states is no longer unrealistic. Thanks to advances in digital technology and analytic techniques, tools now exist to track digital footprints and make informed predictions about consumers’ feelings and emotions in real time. This capability to assess consumers’ psychological states presents contemporary marketers with opportunities to customize marketing content to address consumers’ immediate psychological needs. Context-aware recommendation systems, for instance, already leverage consumers’ mood or emotions to enhance recommendations for music or movies. These advancements signify a shift towards more personalized and targeted marketing strategies, enabling marketers to connect with consumers on a deeper level by delivering content that resonates with their current psychological state.

Our research illuminates a promising direction: tailoring promotional efforts based on consumers’ perceived levels of personal control. For consumers feeling a strong sense of personal control, marketing campaigns for mystery products should spotlight elements like the enjoyment and thrill of uncertainty. Conversely, when targeting individuals with lower perceived control, the focus should pivot towardss highlighting the array of choice options available, thereby fostering a perception of control. This approach enables consumers to feel empowered in the purchasing process and enhances their appreciation for the uncertainty inherent in mystery products. Building upon this insight, marketers can refine and emphasize other product attributes to cultivate a sense of control in consumers who seek it. For instance, enabling consumers to personally “spin the wheel” to select the destination of their random flight ticket may bolster their sense of personal control and increase their likelihood of making a purchase.

### 10.3. Limitations and Future Research

Current research is not without limitations.

First, the sample used in this study was primarily Chinese. While we anticipate similar results in other cultural contexts, it is essential to acknowledge the possibility of cross-cultural variations. Subsequent studies could incorporate samples from different countries to validate and generalize our findings across diverse cultural backgrounds.

Second, the experimental setting focused solely on random flight tickets in the travel industry, potentially limiting the generalizability of our findings to other industries. Future research could explore consumer responses to mystery products in various sectors, including food and beverage (e.g., McDonald’s) and luxury goods (e.g., LV).

Third, in manipulating personal control, we had a high versus low personal control condition, without a control condition. We decided to not include a neutral condition as it is tricky to define a neutral state in terms of feelings of personal control. For this reason, many of the prior studies exploring the effect of personal control chose to compare only between feelings of high versus low personal control, and discuss their findings as differential effects of high versus low personal control. Likewise, the current findings should be interpreted as relative differences between the states of feeling high personal control versus low personal control, and not as evidence for feelings of high (or low) personal control increasing (or decreasing) the appeal of mystery products compared to a neutral state. This suggests an opening for future research. It would be meaningful to examine a baseline in people’s sense of personal control and further explore the effect of increasing or decreasing it from the baseline. Previous literature indicates that individuals typically perceive themselves to have relatively high level of personal control at baseline (e.g., [34,39,40]). Indeed, in one study with a neutral condition, no significant difference was found between feelings of personal control in the neutral versus high control conditions. This supports the notion that individuals generally maintain a baseline perception of relatively high personal control. Participants’ feelings of personal control were significantly reduced in the low control condition. Interestingly, it was well above midpoint of the scale, just like what we observed in the manipulation check results of our studies [41].

Finally, psychology literature suggests that the sense of control can stem not only from perceptions of personal control, but also from perceptions of predictability of the world at large. Interestingly, depending on its source, the sense of control may have varying effects [32]. Specifically, when low personal control arises from a perception of the world as unpredictable, consumers embrace new choices and exhibit heightened novelty-seeking tendencies. On the other hand, when low personal control stems from a perceived lack of personal control, consumers may tend to avoid new choices, leading to a decrease in novelty-seeking behavior. In the current experiments, we focused solely on personal control. Future research should manipulate the sense of control stemming from unpredictability and explore whether the findings from current research remain the same. This will provide a more comprehensive understanding of both facets of control.

## Figures and Tables

**Table 1:** Moderated mediation analysis of Study 2 results.

Moderator	N	PI (Y)		
		Indirect Effect	LLCI	ULCI
Choice availability (W)	407	−0.77	−1.12	−0.43
No Choice	207	0.79	0.53	1.05
Choice	200	−0.02	−0.19	0.25

The interaction effect of personal control and choice availability on appreciation of uncertainty was significant.

## Data Availability

The data used in the research have been uniquely generated through the scheme of the experiment. It will be available upon request.
